# The Visual Advantage Effect in Comparing Uni-Modal and Cross-Modal Probabilistic Category Learning

**DOI:** 10.3390/jintelligence11120218

**Published:** 2023-11-27

**Authors:** Xunwei Sun, Qiufang Fu

**Affiliations:** 1 State Key Laboratory of Brain and Cognitive Science, Institute of Psychology, Chinese Academy of Sciences, Beijing 100101, China; sunxw@pku.edu.cn; 2 Department of Psychology, University of Chinese Academy of Sciences, Beijing 100083, China; 3 Beijing Key Laboratory of Behavior and Mental Health, School of Psychological and Cognitive Sciences, Peking University, Beijing 100080, China

**Keywords:** weather prediction task, cross-modal learning, implicit learning

## Abstract

People rely on multiple learning systems to complete weather prediction (WP) tasks with visual cues. However, how people perform in audio and audiovisual modalities remains elusive. The present research investigated how the cue modality influences performance in probabilistic category learning and conscious awareness about the category knowledge acquired. A modified weather prediction task was adopted, in which the cues included two dimensions from visual, auditory, or audiovisual modalities. The results of all three experiments revealed better performances in the visual modality relative to the audio and audiovisual modalities. Moreover, participants primarily acquired unconscious knowledge in the audio and audiovisual modalities, while conscious knowledge was acquired in the visual modality. Interestingly, factors such as the amount of training, the complexity of visual stimuli, and the number of objects to which the two cues belonged influenced the amount of conscious knowledge acquired but did not change the visual advantage effect. These findings suggest that individuals can learn probabilistic cues and category associations across different modalities, but a robust visual advantage persists. Specifically, visual associations can be learned more effectively, and are more likely to become conscious. The possible causes and implications of these effects are discussed.

## 1. Introduction

The ability to learn the correspondence between cues and outcomes is crucial for humans (Mishkin et al. 1984). For instance, determining the freshness of vegetables through color and smell, or judging the friendliness of strangers based on their appearances and conversations. Perhaps most people who are not well-versed in these skills may find such categorization judgments challenging. This feeling could mainly be due to the correspondence between cues and results not being deterministic but probabilistic. Specifically, a cue does not definitively point to a specific outcome but corresponds to multiple outcomes with different probabilities. As a result, the relationship between cues and outcomes becomes more ambiguous and intricate. However, people can still learn from probabilistic cues, and this cognitive ability is known as probabilistic category learning (Knowlton et al. 1994).

The weather prediction (WP) task is a typical paradigm for studying probabilistic category learning (e.g., Knowlton et al. 1994, 1996a). Generally, in the WP task, participants are presented with four different cues, each with a certain probability for sunny or rainy weather. The combination of cues in each trial may consist of one to four cues. In the beginning, participants can only make guesses. After making a prediction, they would receive feedback on whether it is correct. Through this process, participants could gradually learn the association between cues and outcomes (Kalra et al. 2019; Knowlton et al. 1994).

Initially, the early stage of the WP task was believed to rely on the implicit procedural learning system primarily. This perspective was in line with the multiple systems view of learning, which assumes that there are several learning systems, such as the explicit learning system, processed explicitly or consciously and supported by the hippocampus, and the implicit learning system, processed implicitly or unconsciously and supported by the striatum (Ashby et al. 1998; Green and Shanks 1993; Hayes and Broadbent 1988). The view that the WP task recruited mainly implicit processes has been supported by several empirical studies. Parkinson’s patients (procedural deficit) showed impaired learning performance in the early stages of the training phase compared to healthy participants (Knowlton et al. 1996a). In comparison, amnesic patients (declarative deficit) performed as well as healthy individuals in the early stages of the training phase (Knowlton et al. 1994). Neural imaging studies further indicate a significant deactivation in the medial temporal lobe during the early stages of learning (Poldrack et al. 1999). These results suggested that an implicit rather than explicit memory system is recruited in the early stages of the WP task. Recently, the WP task has continued to be a standard paradigm for assessing implicit procedural learning (Baron and Arbel 2022; Gabay and Goldfarb 2017; Kalra et al. 2019).

However, some studies suggest that the WP task may mainly involve explicit learning processes. For example, it was found that participants acquired explicit knowledge of the task structure and judgment strategy during probabilistic category learning (Newell et al. 2007). In addition, manipulation that disrupted explicit learning reduced accuracy, but manipulation that disrupted implicit learning made no difference to accuracy (Price 2009). For example, performing a memory task simultaneously could dampen feedback-based WP task performance (Lagnado et al. 2006; Newell et al. 2007). These results indicate that the implicit procedural system may not dominate WP task learning.

Moreover, a recent study used the “pattern analyses” method to analyze participants’ learning strategies and found that they likely rely on flexible strategies (Bochud-Fragnière et al. 2022). Consistently, the functional Magnetic resonance imaging (fMRI) results also indicate that the WP task involves both implicit and explicit learning systems, with the explicit system being active in the early stages of learning (Hopkins et al. 2004; Poldrack et al. 2001). Thus, we assume that completing WP tasks should be viewed as a process involving multiple learning systems. The question is to identify which factors would influence the weight of involvement of the different systems.

Moreover, another critical but overlooked question is how people perform when probabilistic category learning involves multiple sensory cues. People commonly rely on multisensory information for making probabilistic category judgments. Taking weather forecasting as an example, experienced individuals rely on visual cues, such as the sky and clouds, tactile cues, such as humidity and wind direction, and gustatory cues, such as the taste of the air, to make weather predictions. However, existing research has mainly focused on visual cues, such as pictures (e.g., Kemény and Lukács 2009; Knowlton et al. 1994), tarot cards (e.g., Gluck et al. 2002), and geometric shapes (e.g., Kalra et al. 2019; Kéri et al. 2002). It remains unclear whether the cue modality influences the performance in probabilistic category learning tasks and, further, the recruitment of implicit and explicit learning systems.

The importance of multi-modality lies not only in the composition of our environment, but also in the benefits it provides us. (Murray et al. 2016; Shams and Seitz 2008). Essentially, consistent rather than conflicting multisensory information is more reliable and more likely to capture people’s attention (Bahrick et al. 2004; Broadbent et al. 2018; Yildirim and Jacobs 2012). Therefore, in terms of perception, a multisensory environment is beneficial for detection and discrimination (Evans 2020; Marchant et al. 2012; von Saldern and Noppeney 2013). Furthermore, in terms of higher cognitive processing, multisensory information can facilitate memory, decision-making, and learning (Eördegh et al. 2019; Matusz et al. 2017; Raposo et al. 2012; Wu et al. 2021). Thus, one may assume that the cue modality can influence the performance in probabilistic category learning and, further, the recruitment of different learning systems.

The current study aimed to investigate how cues from different modalities may lead to differences in learning performance and the varying degrees of involvement of the implicit and explicit learning processes or systems. To explore the effects of modality on learning, we incorporate auditory and visual cues in a modified WP task, in which two cues from auditory modality, visual modality, or audiovisual modalities were adopted. To investigate the involvement of implicit and explicit processes or systems in learning, we used two explicit tests. As for the performance of different modalities, there have been inconsistent findings in previous research. On the one hand, category learning and associative learning literature suggest that cross-modality often has advantages over unimodality when cross-modal information is redundant (e.g., Wu et al. 2021) or the when learning from stimuli in different modalities is conducted separately (Eördegh et al. 2019). On the other hand, the detection task has shown that, when stimuli of different modalities are presented rapidly, participants often respond more to the visual component than the auditory component, known as the Colavita visual dominance effect (e.g., Colavita and Weisberg 1979; Koppen et al. 2009). Therefore, if learning can benefit from multisensory information, we expected that there would be a cross-modal advantage in the current study. However, considering that the cross-modal information in the current study is not redundant, and the stimuli of different modalities are presented in mixed and continuous succession, if the participants can process visual stimuli more effectively, we expected that there would be a visual advantage effect.

To preview the results, we found a visual advantage effect, i.e., the performance was best under the visual modality. Moreover, participants primarily acquired unconscious knowledge in the audio and audiovisual modalities but conscious knowledge in the visual modality. We manipulated various potential influencing factors to explore whether the visual advantage effect was robust. All factors were unable to eliminate the visual advantage effect on learning performance. However, the factors increased explicit knowledge under auditory and audiovisual modalities.

## 2. Experiment 1

Experiment 1 aimed to investigate whether the cue modality (visual, auditory, or audiovisual) can influence the knowledge about probability categories acquired in the WP task; specifically, the relationship between cues and outcomes. To guarantee comparability between cues of different modalities, we included two cues in each trial and ensured that the prediction probability of the two cues was 0.9. Moreover, we aimed to explore whether category knowledge can be acquired unconsciously. To measure the explicitness of the acquired knowledge, we utilized two consciousness level tests: one was the post-decision wagering task during training (Persaud et al. 2007), and the other was the adapted process dissociation procedure (PDP) after training (Destrebecqz and Cleeremans 2001; Jacoby 1998). As in the previous studies, if the explicit test results revealed that participants acquired some conscious knowledge, it would indicate that explicit learning systems or processes contributed to the learning performance.

Previous research has shown that the early and late stages of learning involve different learning systems (Fu et al. 2008), and learning trajectories differ across modalities, but the learning outcomes are identical (Kemény and Lukács 2013a). Therefore, we manipulated the amount of training in two separate experiments. In Experiment 1a, the training phase was relatively short, consisting of 120 trials; in Experiment 1b, the training phase was rather long, including 360 trials. Based on previous studies, we predicted that participants acquire partial conscious knowledge when the training phase was longer in Experiment 1b rather than in Experiment 1a, while the stimulus modality would influence learning performance in Experiment 1a but not in Experiment 1b.

### 2.1. Methods

#### 2.1.1. Participants

We conducted an a priori power calculation using G*Power 3.1 for repeated ANOVA measures with effect size f = 0.3, α = 0.05, and (1 − β) = 0.8, which suggested a sample size of 20 (Faul et al. 2009). Our targeted sample size was at least 20 for all the experiments. In Experiment 1a, 27 university students (15 females, *M_age_* = 24.6 ± 2.3 years) voluntarily participated. All participants had normal hearing and vision (or corrected vision) and had not previously participated in similar experiments. Participants received monetary incentives based on their participation duration. According to the performance in the test phases, four participants who performed at the chance level (50%) in all three modalities were excluded. Additionally, two participants with scores beyond three standard deviations from the mean were excluded. The data from the remaining 21 participants were included in the data analysis.

In Experiment 1b, 29 university students (15 females, *M_age_* = 21.83 ± 1.98 years) voluntarily participated. Two participants were excluded from the analysis due to their test performance being more than three standard deviations away from the mean. The data from the remaining 27 individuals were included in the analysis.

#### 2.1.2. Materials

**Figure 1:**
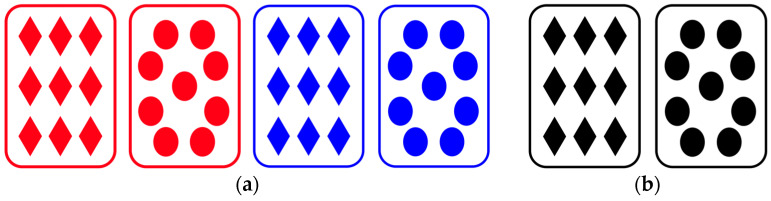
Examples of visual stimuli in Experiment 1. (**a**) The red and blue pictures were stimuli for the visual modality. (**b**) The black-and-white pictures were stimuli for the audiovisual modality.

Each trial consisted of two cues: color (red, blue) and shape (circle, rhombus) in the visual modality, timbre (Guitar, Wooden fish) and pure tones (high, low) in the auditory modality, and shape and timbre in the audiovisual modality. Specifically, visual cues were cards with nine small geometric figures, as shown in Figure 1. The timbre cues were generated using GarageBand. Pure tones were created using Adobe Audition, with 600 Hz for high pure tones and 200 Hz for low pure tones. The auditory cues lasted for 2000 ms, in which timbre and pitch stimuli were alternately presented twice, with the first sound appearing at the beginning and subsequent sounds appearing at intervals of 500 ms, with each lasting approximately 400 ms. For the audiovisual cue, shape stimuli were black-and-white images without color, and timbre stimuli were instrumental sounds presented four times.

The combination of cues and the corresponding relationship between cues and outcomes are shown in Table 1. There were 12 cue combinations, and all combinations had a predictive power of 90%. Meanwhile, the probability of each specific cue for sunny or rainy weather was 50%. For example, the red–circle combination predicted sunny weather with a probability of 90%, but the red–rhombus combination predicted rainy weather with a probability of 90%. Thus, the probability of red color corresponding to either weather outcome was 50%. This design ensured that participants had to combine two cues to make accurate predictions.

#### 2.1.3. Procedure and Data Processing

The experimental procedures in this study were programmed and run using E-prime 2.0 (Psychology Software Tools, Inc. 2007). Stimuli were presented on a Philips CRT 22-inch (36 cm × 28 cm) monitor with a refresh rate of 100 Hz and a 1024 × 768 pixels resolution. The participants were maintained approximately 60 cm from the screen and wore headphones.

**Figure 2:**
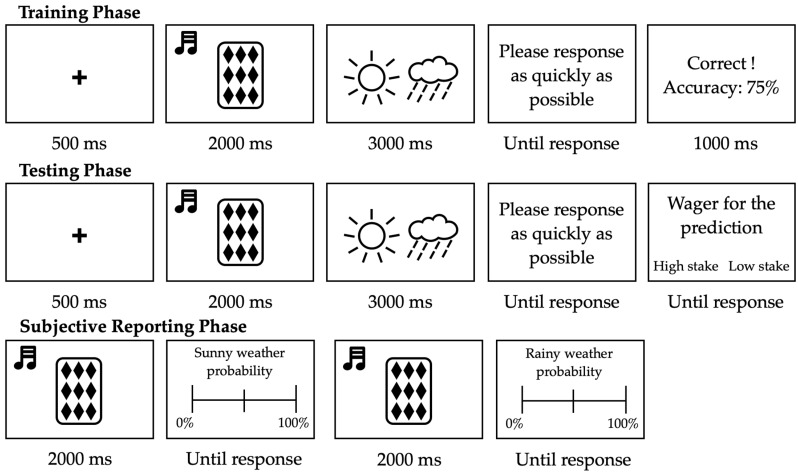
The procedure of a single trial in Experiment 1.

The experiment consisted of three phases: the training phase, the testing phase, and the subjective reporting phase. The trial procedure in each phase is illustrated in Figure 2. In the training phase, participants were required to learn the correspondence between cues and weather outcomes based on feedback. Each trial presented a cue combination after a fixation point. Participants observed the stimulus and predicted the outcome. They could only start pressing the keys when the weather image was displayed, with “F” representing the rainy weather and “J” the sunny weather (counter-balanced across participants). If participants did not press a key within three seconds, a prompt saying “Please respond as soon as possible” would be presented until they responded. Subsequently, feedback on accuracy and cumulative accuracy were displayed. The training phase consisted of 5 blocks, each with 24 trials, for a total of 120 trials, with each stimulus combination randomly presented 10 times.

In the testing phase, participants did not receive feedback after making predictions but instead were asked to place bets on their predictions. This is known as post-decision wagering. If their prediction was correct, they would win the corresponding bet. Otherwise, they would lose it. The testing phases consisted of 120 trials, with each stimulus combination randomly presented 10 times.

After the testing phase, there was a subjective reporting phase, where participants went through three rounds. In each round, all 12 combinations were presented in a random order. In each round, a combination was presented, and participants needed to report the probability of sunny weather, cloudy weather, or the importance of each dimension in the combination. Thus, the subjective reporting phase consisted of 36 trials.

#### 2.1.4. Data Processing

Due to the limitations of null hypothesis significance testing in determining the likelihood of supporting the null hypothesis, we calculated the Bayes factor when the *p* value was less than 0.05. The Bayes factor was calculated using the default settings in JASP (Wagenmakers et al. 2018). We reported evidence of an alternative model compared to the null model, and interpreted a Bayes factor (*BF_10_*) of 0 to 1/3 as evidence for the null hypothesis, 1/3 to 3 indicating insensitivity to either hypothesis, and above 3 as evidence for the alternative hypothesis (Dienes and McLatchie 2018).

### 2.2. Results

#### 2.2.1. Results for Experiment 1a

**Figure 3:**
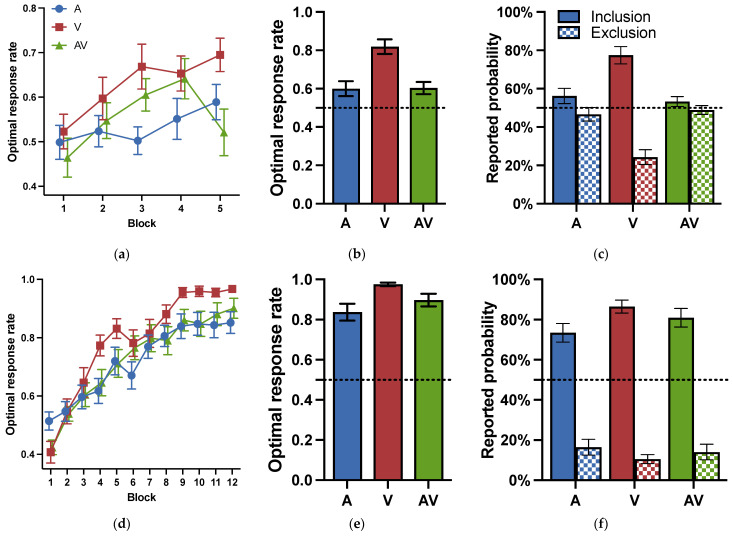
Optimal response rates and reported probabilities for Experiment 1. (**a**) Optimal response rates for the training phase in Experiment 1a. 1 block = 24 trials; (**b**) optimal response rates for the testing phase in Experiment 1a; (**c**) reported probability of cue combinations in Experiment 1a; (**d**) optimal response rates for the training phase in Exp. 1b. 1 block = 30 trials; (**e**) optimal response rates for the testing phase in Experiment 1b; (**f**) reported probability of cue combinations in Experiment 1b. A: audio modality; V: visual modality; AV: audiovisual modality. Error bars reflect standard errors.

The optimal response rates for each block during the training phase are shown in Figure 3a. A two-way repeated analysis of variance (ANOVA) for cue modality (visual, auditory, audiovisual) and block (1–5) was conducted to examine whether the cue modality influenced the learning effect during the training phase. The results revealed that the main effects of block were significant: *F* (4, 80) = 4.449, *p* = .003, $\eta_{p}^{2}$ = 0.182, indicating a learning effect. The main effects of modality were significant: *F* (2, 40) = 6.036, *p* = .005, $\eta_{p}^{2}$ = 0.232, suggesting that there were differences in learning between modalities. Post-hoc tests (Holm corrected) showed that the participants performed better in the visual modality than in the other two modalities: *ts* (20) > 2.522, *ps* < 0.032, *ds* > 0.364. The optimal response rates had no difference between the auditory and audiovisual modalities: *t* (20) = 0.809, *p* = .423, *d* = 0.117, *BF* = 0.150. The interaction between modality and blocks was insignificant: *F* (8, 160) = 1.109, *p* = .360, $\eta_{p}^{2}$ = 0.053, *BF* = 0.142.

The results for the testing phase in Experiment 1a are shown in Figure 3b. To examine whether the participants learned category knowledge, we conducted *t*-tests to compare the optimal response rates under each modality to the chance level. The results revealed that the optimal response rates under all three modalities were significantly above the random level: *ts* (20) > 2.527, *ps* < 0.020, *ds* > 0.552. The results suggested that participants under all three modalities acquired some category knowledge.

To investigate the impact of cue modalities on probabilistic category learning during the testing phase, a one-way repeated measures ANOVA was conducted for cue modality (visual, auditory, audiovisual). The results showed that the main effects of cue modality were significant: *F* (2, 60) = 11.354, *p* = .007, $\eta_{p}^{2}$ = 0.275. The results of the post-hoc test (Bonferroni corrected) revealed that the participants performed significantly better under the visual modality (*M* = 0.819, *SD* = 0.040) than the auditory modality (*M* = 0.600, *SD* = 0.039) and the audiovisual modality (*M* = 0.603, *SD* = 0.032), *ts* (20) > 4.016, *ps* < 0.001, *ds* > 1.217. There was no significant difference between the auditory and audiovisual modalities: *t* (20) = 0.068, *p* = 1.000, *d* = 0.021, *BF* = 0.304. The results confirmed that the performance in category learning was the highest under the visual modality, demonstrating the advantage effect of the visual modality.

For the post-decision wagering, we used the binomial test to investigate whether the participants acquired explicit or conscious knowledge. No significant difference from the random level (50%) would indicate that the participants were unaware of whether their responses were correct or incorrect after each judgment, and they acquired unconscious knowledge. Alternatively, a significant difference, would suggest that the participants were aware of the correctness of their responses and acquired a certain level of explicit or conscious knowledge. The results showed that the proportion of high bets in correct trials is 78% (537/688), significantly higher than the chance level (*p* < .001) under the visual modality, while the proportion of low bets in incorrect trials is 40% (61/152), significantly lower than the chance level (*p* = 0.02). However, under auditory and audiovisual modalities, participants made neither more high bets to increase gains in correct trials, nor more low bets to reduce losses in incorrect trials (*ps* > 0.219). The results indicated that participants under the visual modality were conscious of their decisions when they classified correctly or incorrectly, while participants under the auditory and audiovisual modalities were not.

The results of the subjective reporting test in Experiment 1a are shown in Figure 3c. Participants were required to report the prediction probability of sunny and rainy weather separately for each cue combination. If participants obtained perfect explicit knowledge, the cue combination with a probability of 90% of sunny outcome should be reported with 90% of sunny under the sunny instructions, and should be reported with 10% of rainy under the rainy instructions. Or else, if participants acquired no explicit knowledge, there would be no significant differences between the different instructions for the same cue combination. A paired t-test indicated that, under the visual modality, there was a significant difference between the different instructions *t* (20) = 6.746, *p* < .001, and *d* = 1.472. However, under the auditory and audiovisual modalities, there was no significant difference between them, so *ts* (20) < 1.404, *ps* > 0.176, *ds* < 0.306. The results confirmed the acquisition of conscious knowledge under the visual modality and the acquisition of unconscious knowledge under auditory and audiovisual modalities.

#### 2.2.2. Results for Experiment 1b

The optimal response rates for each block during the training phase in Experiment 1b are shown in Figure 3d. A two-way repeated ANOVA for cue modality (visual, auditory, audiovisual) and block (1–12) was conducted to examine whether the learning effect was influenced by the cue modality during the training phase. The results revealed that the main effects of block were significant: *F* (11, 286) = 64.806, *p* < .001, $\eta_{p}^{2}$ = 0.714, indicating a learning effect. The main effects of modality were significant: *F* (2, 52) = 5.921, *p* = .005, $\eta_{p}^{2}$ = 0.185. Post-hoc tests showed that the participants performed better in the visual modality than in the other two modalities: *ts* (26) > 2.522, *ps* < 0.019, *ds* > 0.352, while there was no difference between the auditory and audiovisual modalities: *t* (26) = 0.517, *p* = .607, *d* = 0.068, *BF* = 0.121. There was also a significant interaction effect between modality and block: *F* (22, 572) = 1.832, *p* = .012, $\eta_{p}^{2}$ = 0.066. The results revealed a visual advantage effect when the training phase was long.

Figure 3e shows the optimal response rates in the testing phase in Experiment 1b. To examine whether participants learned category knowledge, a one-sample *t*-test was conducted comparing the optimal response rates with the chance level. The results indicated that the optimal response rates under all three modalities were significantly higher than the chance level: *ts* (26) > 8.042, *ps* < 0.001, *ds* > 1.582. The results suggested that participants in all three modalities acquired category knowledge.

To investigate the impact of cue modalities on probabilistic category learning, a one-way repeated ANOVA was conducted for the factor of modality (visual, auditory, audiovisual). The results revealed that the main effect of modality was significant: *F* (2, 78) = 5.045, *p* = .009, $\eta_{p}^{2}$ = 0.115. Post-hoc tests showed that the optimal response rate in the visual modality (*M* = 0.975, *SD* = 0.047) was significantly higher than in the auditory modality (*M* = 0.837, *SD* = 0.218), *t* = 3.168, *p* = .007, *d* = 0.876). There was no significant difference between the visual modality and the audiovisual modality (*M* = 0.897, *SD* = 0.165): *t* = 1.786, *p* = .234, *d* = 0.642, *BF* = 2.582, or between the auditory and audiovisual modalities: *t* = 1.382, *p* = .513, *d* = 0.312, *BF* = 0.471. The results suggested that the visual advantage remained after a relatively long training phase.

For the post-decision wagering, binomial tests revealed that, under the visual modality, the proportion of high bets in correct trials was 91% (991/1093), significantly higher than the chance level (*p* < .001), but the proportion of low bets in incorrect trials was 52% (14/27), which did not significantly differ from the chance level (*p* = .999). Under the auditory modality, the proportion of high bets in correct trials was 76% (722/944), significantly higher than the chance level (*p* < .001), and the proportion of low bets in incorrect trials was 78% (137/176), also significantly higher than the chance level (*p* < .001). Under the audiovisual modality, the proportion of high bets in correct trials was 87% (875/1009), significantly higher than the chance level (*p* < .001), and the proportion of low bets in incorrect trials was 77% (85/111), significantly higher than the chance level (*p* < .001). The results indicated that all participants acquired some conscious knowledge.

The subjective reporting results in Experiment 1b are shown in Figure 3f. The results of the paired *t*-test further revealed that under all three modalities, there were significant differences between sunny and rainy instructions: *ts* (26) > 7.762, *ps* < 0.001, *ds* > 1.494. Consistently, the results suggested that participants were able to consciously distinguish which cue combinations predicted sunny and cloudy weather under all three modalities.

### 2.3. Discussion

The results of Experiment 1a indicated that, under a short amount of training (120 trials), participants could make relatively accurate predictions about the weather by combining auditory and visual information; further, they could obtain both unimodal and cross-modal categorical knowledge. The performance under the visual modality was significantly higher than that under the auditory and audiovisual modalities, indicating a visual advantage effect. Additionally, participants under the visual modality acquired some conscious knowledge, while participants under the auditory and audiovisual modalities primarily acquired unconscious knowledge. These findings demonstrate that the cue modality influences both performance and the explicitness of the acquired knowledge in probabilistic category learning.

The visual advantage observed in Experiment 1a may be because the two visual cues can be processed simultaneously, while the two auditory cues need to be processed sequentially. In this way, it might be easier to integrate the visual cues than the auditory cues, resulting in a faster improvement in accuracy under visual modalities. If the training phase can be extended, the differences in accuracy between the visual and auditory modalities may be eliminated. To examine this possibility, we extended the training phase in Experiment 1b from 120 trials to 360 trials. The results indicated that, although the optimal response rates improved in all three modalities, the visual advantage persisted. Additionally, participants in all three modalities acquired primarily conscious knowledge with sufficient learning. That is, the amount of training influenced the acquisition of conscious knowledge but did not change the visual advantage effect.

The other possible explanation for the visual advantage effect might be that the visual stimuli were easier to name than auditory stimuli. For example, a pure tone of 600 Hz was relatively tricky to label compared with the circle shape. This may lead to the visual stimuli more easily to be remembered than the audio stimuli. Therefore, we designed Experiment 2 to further explore the visual advantage effect by increasing the difficulty of naming visual cues.

## 3. Experiment 2

Considering that the visual stimuli in Experiment 1 might be easier to name and identify than auditory stimuli, Experiment 2 adopted complex visual stimuli that were more difficult to name and identify. The aim of Experiment 2 was to investigate whether the advantage of the visual modality persisted when the visual stimuli became more complex to label. Moreover, the feedback was visually presented, which might lead to a priming effect on visual trials. To investigate whether the feedback had a priming effect on subsequent learning, Experiment 2a still utilized visual feedback, while Experiment 2b used auditory feedback. If the complexity of visual stimuli reduces visual learning performance, the visual advantage effect might disappear. Further, if the modality of feedback reduces visual learning performance, the visual advantage effect might also disappear.

### 3.1. Methods

#### 3.1.1. Participants

A total of 26 undergraduate and graduate students (15 females, *M_age_* = 22.44 ± 2.03) participated in Experiment 2a. All participants voluntarily participated in the experiment, had normal hearing and vision (or corrected vision), and had not previously participated in similar experiments. Participants received monetary incentives based on their participation duration. According to the performance in the test phase, three participants who performed at chance level (50%) were excluded, and one participant who experienced a malfunction in the experimental procedure was also excluded. The data from the remaining 22 participants were included in the data analysis.

A total of 30 undergraduate and graduate students (16 females, *M_age_* = 21.62 ± 2.53) took part in Experiment 2b. Based on the performance in the test phase, three participants who performed at a chance level (50%) were excluded, and three participants who experienced a malfunction in the experimental procedure were also excluded. The data of the remaining 24 participants were included in the data analysis.

#### 3.1.2. Materials

**Figure 4:**
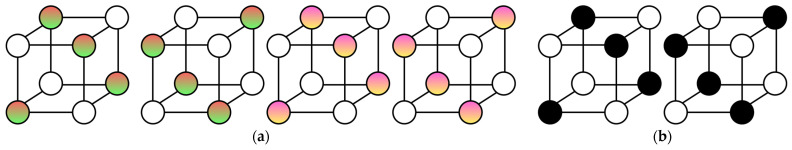
Examples of stimuli in the visual modality in Experiment 2. (**a**) The colored pictures were stimuli for the visual modality; (**b**) The black-and-white pictures were stimuli for the audiovisual modality.

In Experiment 2, the auditory stimuli remained the same as in Experiment 1, while all the visual stimuli were new and more complex shapes (see Figure 4). The shape dimension was changed from geometric shapes to cubic shapes. The eight vertices of the cube were represented by circles, with four of them colored. The different positions of the colored vertices formed two different cube shapes. Additionally, the color dimension was changed from solid colors to gradient colors, with two levels: red–green and yellow–purple.

#### 3.1.3. Procedure

The procedure in Experiment 2 was similar to that in Experiment 1, except that, in the training phase of Experiment 2b, the visual feedback was replaced with auditory feedback. In other words, the participants were informed about their predicted results’ correctness through speech instead of visual characters. Both the training and testing phases consist of 120 trials. Forty trials each for visual, auditory, and audiovisual modalities. The order of presentation was randomized.

### 3.2. Results

#### 3.2.1. Experiment 2a

**Figure 5:**
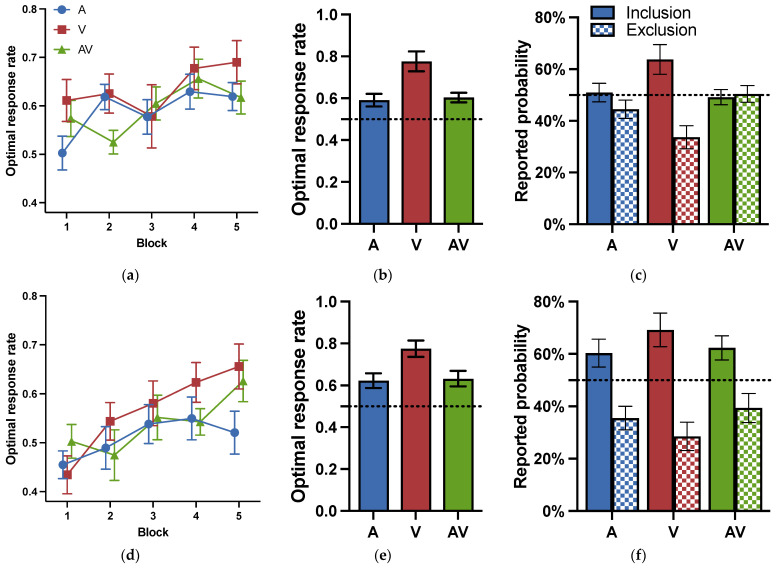
Optimal response rates and reported probabilities for Experiment 2. (**a**) Optimal response rates for the training phase in Experiment 2a. 1 block = 24 trials; (**b**) optimal response rates for the testing phase in Experiment 2a; (**c**) reported probability of cue combinations in Experiment 2a; (**d**) optimal response rates for the training phase in Experiment 2b. 1 block = 24 trials; (**e**) optimal response rates for the testing phase in Exp. 2b; (**f**) reported probability of cue combinations in Experiment 2b. A: audio modality; V: visual modality; AV: audiovisual modality. Error bars reflect standard errors.

The optimal response rates for each block in the training phase are shown in Figure 5a. A two-way repeated ANOVA for cue modality (visual, auditory, audiovisual) and block (1–5) was conducted to examine whether the learning effect was influenced by the cue modality. It revealed that the main effects of block were significant: *F* (4, 84) = 3.092, *p* = .020, $\eta_{p}^{2}$ = 0.128, indicating a learning effect. Neither the main effect of modality, *F* (2, 42) = 1.343, *p* = .272, $\eta_{p}^{2}$ = 0.060, *BF* = 0.183, nor the interaction effect between block and modality, *F* (8, 168) = 0.964, *p* = .466, $\eta_{p}^{2}$ = 0.044, *BF* = 0.068, reached significance.

Figure 5b shows the optimal response rates in the testing phase in Experiment 2a. To examine whether the participants learned category knowledge, we conducted one-sample *t*-tests to compare the optimal response rates to the chance level. The performance in all three modalities was significantly higher than the chance level: *ts* (21) > 2.863, *ps* < 0.009, *ds* > 0.610. The results suggested that participants did acquire some category knowledge under all three modalities.

To investigate the impact of cue modalities on probabilistic category learning, a one-way repeated ANOVA was conducted for the factor of modality (visual, auditory, audiovisual). The main effects of modality were significant: *F* (2, 63) = 7.979, *p* < .001, $\eta_{p}^{2}$ = 0.202). Post-hoc analyses indicated that the optimal response rate for the visual modality (*M* = 0.776, *SD* = 0.233) was significantly higher than that for the auditory modality (*M* = 0.591, *SD* = 0.149) and the audiovisual modality (*M* = 0.603, *SD* = 0.111): *ts* > 3.333, *ps* < 0.004, *ds* > 0.947, *BFs* > 12.486. However, there was no significant difference between the auditory and audiovisual modalities: *t* = 0.241, *p* = 1.000, *d* = 0.095, *BF* = 0.310. The results indicated that the visual advantage effect persisted.

For the post-decision wagering, the binomial tests revealed that, under the visual modality, the proportion of high bets in correct trials was 81% (551/683), which was significantly higher than the chance level (*p* < .001), while the proportion of low bets in incorrect trials was 50% (98/197), which did not differ significantly from random levels (*p* = .913). Under the auditory modalities, the proportion of high bets in correct trials was 51% (238/468), and the proportion of low bets in incorrect trials was 50% (183/360). Both proportions did not differ significantly from the chance level (*ps* > 0.059). Under the audiovisual modality, the proportion of high bets in correct trials was 45% (239/531), and the proportion of low bets in incorrect trials was 40% (139/349). Both proportions were significantly lower than the chance level (*ps* < 0.024). The results indicated that the participants were aware of the correctness of their responses under the visual modality but were not under the other modalities.

The subjective reporting results in Experiment 2b are shown in Figure 5c. Paired *t*-tests revealed that there was a significant difference in the subjective report probabilities between sunny and rainy instructions in the visual modality: *t* (21) = 2.922, *p* = .008, and *d* = 0.623. However, there was no significant difference between the different instructions in the auditory and audiovisual modalities: *ts* (21) < 0.947, *ps* > 0.198, *ds* < 0.202, *BFs* < 0.333. Consistently, the results indicated that participants under the visual modality could distinguish which combinations predicted sunny weather and which predicted cloudy weather, but participants in the auditory and audiovisual modalities could not.

#### 3.2.2. Experiment 2b

The optimal response rates for each block in Experiment 2b are shown in Figure 5d. A two-way repeated ANOVA for modality (visual, auditory, audiovisual) by block (1–5) was conducted to examine if the learning effect was influenced by the cue modality during the training phase. It revealed that the main effects of block were significant: *F* (4, 92) = 5.520, *p* < .001, $\eta_{p}^{2}$ = 0.194, proving a learning effect. Neither the main effect of the modality, *F* (2, 46) = 1.852, *p* = .168, $\eta_{p}^{2}$ = 0.075, *BF* = 0.220, nor the interaction between the block and modality, *F* (8, 184) = 1.057, *p* = .395, $\eta_{p}^{2}$ = 0.044, *BF* = 0.078, reached significance.

The optimal response rates during the testing phase in Experiment 2b are shown in Figure 5e. To examine whether the participants learned category knowledge, we conducted one-sample *t*-tests to compare the optimal response rates to the chance level. The performance in all three modalities was significantly higher than the chance level, *ts* (23) > 3.483, *ps* < 0.002, *ds* > 0.711. These results indicated that participants under all modalities had acquired categorical knowledge.

To investigate the impact of cue modalities on probabilistic category learning, a one-way ANOVA was conducted for the factor of modality (visual, auditory, audiovisual). It showed a significant main effect of cue modality: *F* (2, 69) = 5.345, *p* = .007, $\eta_{p}^{2}$ = 0.134. Post-hoc tests revealed that the optimal response rate in the visual modality (M = 0.775, SD = 0.038) was significantly higher than in the auditory modality (*M* = 0.623, *SD* = 0.035), and the audiovisual modality (*M* = 0.632, *SD* = 0.037): *ts* > 2.737, *ps* < 0.024, *ds* > 0.774. There was no significant difference between the auditory and audiovisual modalities: *t* = 0.180, *p* = 1.000, *d* = 0.053. *BF* = 0.291. The results suggested that the visual modality still exhibited an advantage.

For the post-decision wagering, binomial tests revealed that, under the auditory modality, the proportion of high bets in correct trials was 46% (277/478), while the proportion of high bets in incorrect trials was 52% (189/362). Under the audiovisual modality, the proportion of high bets in correct trials was 52% (318/607), while the proportion of low bets in incorrect trials was 51% (179/353). These proportions were not significantly different from the chance level (*ps* > 0.079), indicating that participants were unaware of which trials were correct or incorrect in these two modalities. By contrast, under the visual modality, the proportion of high bets in correct trials was 74% (548/744), significantly higher than the random level (*p* < .001), while the proportion of high bets in incorrect trials was 55% (118/216), not significantly different from the random level (*p* = .196), suggesting that participants were aware of their correct predictions under this modality.

The subjective reporting results of Experiment 2b are shown in Figure 5f. The paired *t*-test revealed significant differences between the sunny and rainy instructions under all three modalities, *ts* (23) > 2.375, *ps* < 0.026, *ds* > 0.485. The results indicated that participants might be consciously aware of which trials predicted sunny or cloudy weather under all three modalities.

### 3.3. Discussion

Experiment 2a adopted complex visual stimuli and visual feedback. We found that there was still a visual modality advantage, and the optimal response rate did not decrease. Additionally, participants primarily acquired conscious knowledge under the visual modality, but participants primarily acquired unconscious category knowledge under the auditory and audiovisual modalities.

Experiment 2b utilized complex visual stimuli and auditory feedback. The results showed that the accuracy during the testing phase was higher under the visual modality than under the other two modalities, indicating the constant presence of visual superiority. However, participants under all modalities expressed some conscious knowledge in the subjective reporting tests. The auditory feedback did not significantly affect the optimal response patterns, but it allowed participants to gain more conscious knowledge under the auditory and audiovisual modalities. Nonetheless, this conscious improvement was limited as participants failed to exhibit a higher stake on correct trials in the auditory and audiovisual modalities than they did in the visual modality.

The results of Experiment 2 demonstrate that increasing the complexity of visual stimuli does not impact visual advantage. One possible explanation might be that the two visual cues belonged to a single object, while auditory stimuli consisted of two objects (pure sound and instrument stimuli), and so did audiovisual stimuli (a visual object and a sound). To investigate the influence of the number of objects the two cues belonged to, we designed Experiment 3, in which the two auditory cues belonged to a single object as the two visual cues.

## 4. Experiment 3

The results of Experiment 2 revealed that neither complex visual stimuli nor auditory feedback could alter the accuracy patterns under the three modalities, thus being unable to change the visual advantage effect. In addition to the factors already explored, the visual advantage effect may be attributed to the differences in object consistency between the two dimensions. Specifically, in Experiments 1 and 2, in the visual modality, color and shape belonged to the same object: the card. In the auditory modality, the two dimensions were divided into instrument and pure tone audios, which belonged to two objects. In the audiovisual modality, the two dimensions were also divided into a card and an instrument audio, falling into different objects. Compared to a unified object, when cues belong to two objects, switching or integrating cues might require more attention shifts and cognitive resources. This may affect the optimal response rate and hinder the formation of conscious knowledge. To address this issue, we recreated the auditory stimuli involving two cues of the same object. Sounds with varying pitches were adopted in Experiment 3. Like visual stimuli, the two dimensions of auditory stimuli (timbre and pitch) belonged to the same object. We expected that unified auditory stimuli might improve the optimal response rate or the acquisition of conscious knowledge in the auditory modality, and thus, the visual advantage effect might disappear.

### 4.1. Methods

#### 4.1.1. Participants

A total of 25 local university students and graduate students (13 females, *M_age_* = 21.37 ± 2.11 years) voluntarily participated in the experiment. All participants had normal hearing and vision (or corrected vision) and had not previously participated in similar experiments. Participants received monetary incentives based on their participation duration. According to the results of the testing phase, one participant who had no significant difference in performance from the chance level (50%) and one participant whose total score was outside of three standard deviations were excluded. The data for the remaining 23 participants were included in the data analysis.

#### 4.1.2. Materials

The visual stimuli for Experiment 3 were the same as those in Experiment 2a. The new auditory stimuli were created using GarageBand. There were two cues: timbre (trombone, guitar) and pitch (C3, C4). Each level of the two cues was combined to form four auditory stimuli. The duration of each auditory stimulus was 2000 ms. The audiovisual stimuli consisted of C3 audio with different timbres and two black-and-white stereograms. It is worth noting that, unlike unified auditory stimuli, the two cues of audiovisual stimuli were not integrated into a single object, but rather belonged to two distinct objects.

#### 4.1.3. Procedure and Data Processing

The experimental procedure was identical to Experiment 2a.

### 4.2. Results

**Figure 6:**
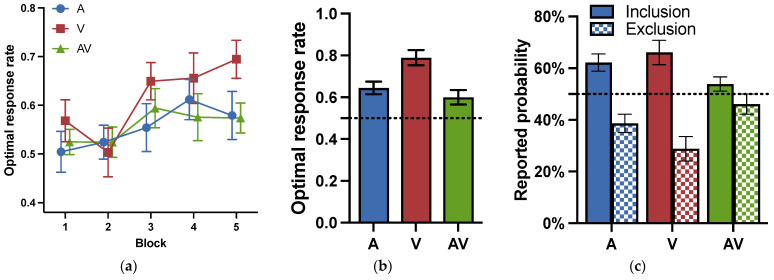
Optimal response rates and reported probabilities for Experiment 3. (**a**) Optimal response rates for the training phase in Exp. 3. 1 block = 24 trials; (**b**) optimal response rates for the testing phase in Experiment 3; (**c**) reported probability of cue combinations in Experiment 3. A: audio modality; V: visual modality; AV: audiovisual modality. Error bars reflect standard errors.

The optimal response rates of each block in the training phase in Experiment 3 are shown in Figure 6a. A repeated ANOVA for modality (visual, auditory, audiovisual) and block (1–5) was conducted to examine whether the learning effect was influenced by the cue modality during the training phase. It revealed that the main effects of block were significant: *F* (4, 88) = 4.460, *p* = 0.002, $\eta_{p}^{2}$ = 0.169, indicating a learning effect. The main effect of modality also reached significance: *F* (2, 44) = 4.068, *p* = .024, $\eta_{p}^{2}$ = 0.156. Post-hoc tests showed that the participants performed better in the visual modality than in the other two modalities: *ts* (22) > 2.383, *ps* < 0.043, *ds* > 0.284. The optimal response rates had no difference between the auditory and audiovisual modalities: *t* (22) = 0.165, *p* = .869, *d* = 0.202, *BF* = 0.105. The interaction between block and modality was insignificant: *F* (8, 176) = 0.606, *p* = .772, $\eta_{p}^{2}$ = 0.027, *BF* = 0.029.

The optimal response rates during the testing phase in Experiment 3 are shown in Figure 6b. To examine whether the participants learned category knowledge, we conducted one-sample *t*-tests to compare the optimal response rates to the chance level. Optimal response rates in all three modalities were higher than the chance level: *ts* (22) > 2.907, *ps* < 0.008, *ds* > 0.606. The results indicated that participants in all three modalities acquired some category knowledge.

To investigate the impact of cue modalities, a one-way repeated ANOVA was conducted for the factor of modality (visual, auditory, audiovisual). It revealed that the main effects modality were significant: *F* (2, 44) = 19.784, *p* < .001, $\eta_{p}^{2}$ = 0.354. Post-hoc tests showed that the optimal response rate in the visual modality (*M* = 0.789, *SD* = 0.173) was significantly higher than those in the other two modalities: *ts* (22) > 4.139, *ps* < 0.001, *ds* > 0.892. There was no significant difference between the auditory (*M* = 0.645, *SD* = 0.144) and audiovisual (*M* = 0.600, *SD* = 0.165) modalities: *t* (22) = 0.721, *p* = .232, *d* = 0.033, *BF* = 0.191. The results demonstrated the presence of a visual modality advantage.

The binomial tests for the post-decision wagering revealed that under the visual modality, the proportion of high bets in correct trials was 77% (560/726), significantly higher than the chance level (*p* < .001), while the proportion of low bets in incorrect trials was 47% (92/216), not significantly different from the chance level (*p* = .335). Similarly, under the auditory modality, the proportion of high bets in correct trials was 64% (381/593), significantly different from the chance level (*p* < .001), while the proportion of low bets in incorrect trials was 53% (172/327), not significantly different from the chance level (*p* = .376). In the audiovisual modality, the proportion of high bets in correct trials was 50% (275/552), and the proportion of low bets in incorrect trials was 53% (196/368), neither of which were significantly different from the random level (*ps* > 0.293). The results indicated that participants acquired some conscious knowledge under auditory and visual modalities, but unconscious knowledge under the audiovisual modality.

The results of the subjective report in Experiment 3 are shown in Figure 6c. The paired *t*-test further revealed that under the visual and auditory modalities; the probabilities were significantly different between sunny and rainy instructions: *ts* (22) > 3.930, *ps* < 0.001, *ds* > 0.819. However, there was no significant difference between them under the audiovisual modality: *t* (22) = 1.279, *p* = .214, *d* = 0.267, *BF* = 0.450. The results confirmed that participants acquired some conscious knowledge under the single-modality modality, but mainly unconscious knowledge under the audiovisual modality.

### 4.3. Discussion

Experiment 3 utilized auditory cues that belonged to one single object and principally replicated the results of Experiment 2b, indicating that the visual advantage effect still existed. Therefore, the number of objects the cues belonged to was not the leading cause of the visual advantage effect. Compared with the accuracy in Experiment 1a, the auditory cues belonging to one object did not improve participants’ accuracy in classification. However, as in Experiment 2b, the new auditory cues enhanced participants’ awareness about the acquired knowledge under the auditory modality but did not influence the acquisition of conscious knowledge under the audiovisual modality. The results suggest that the number of objects that the two cues belonged to could influence the acquisition of conscious knowledge but could not change the visual advantage effect.

## 5. General Discussion

The current research adopted a modified WP task to investigate how cues from different modalities may lead to differences in learning performance and the varying involvement degrees of the implicit and explicit learning systems. The results revealed that the optimal response rate under the visual modality was significantly higher than those under the auditory and audiovisual modalities. Moreover, the conscious awareness of visual category knowledge was also the highest. These results indicated the presence of a visual advantage effect. Interestingly, longer training improved the acquisition of conscious knowledge in all modalities, while auditory feedback and a unified auditory object improved the acquisition of conscious knowledge in the auditory modality. However, these factors could not overturn the visual advantage effect. The results indicated that the cue modality could influence the acquisition of category knowledge and the explicitness of the acquired knowledge in probabilistic category learning.

This study extends the applicability of WP tasks from the previous uni-modality to cross-modalities. Specifically, the results of the audiovisual modality were consistent with previous findings, indicating that participants can integrate information from multiple sensory modalities to categorize cues (Broadbent et al. 2018; Viganò et al. 2021; Yildirim and Jacobs 2015). This is not surprising, as humans rely on information from multiple modalities in daily life to predict the weather or make other inferences (e.g., Stein and Stanford 2008; Stein et al. 2020).

The WP task used in this study incorporates multimodal cues. To ensure comparability between different modalities, we made two modifications to the standard paradigm. First, the original task had 14 different stimulus combinations, with 1–4 cues in each trial (Knowlton et al. 1994, 1996b). In contrast, the combinations in the present study were limited to 12, and each contained two cues. Second, the predictive power of the cues in the original task varied, with some having high (75%) and others having low (57%) predictive power. In contrast, all cue combinations in this study have high predictive power (90%).

The results consistently demonstrate a visual advantage effect, with little significant differences between auditory and audiovisual modalities. We refer to the phenomenon under study as the “visual advantage effect” rather than the “visual dominance effect”. This is because the dominance effect usually refers to a situation where different modalities of information conflict, and one modality takes the upper hand, dominating people’s perceptions (Song et al. 2017; Spence 2009; Witten and Knudsen 2005). However, in this study, the category learning in the three modalities is parallel, and there is no opposing relationship. It is just that the visual modality performs the best.

The visual advantage effect in this study is robust. Experiments 1b, 2a, 2b, and 3, each ruled out the possibilities of the factors the amount of training, the complexity of visual stimuli, modality properties of feedback, and the number of objects the two cues belonged to that could account for the visual advantage effect. Although humans are visual creatures and most information processed by the brain is visual (Pearson 2019), visual processing is not always prioritized. Vision holds the upper hand in determining spatial positions, such as in the ventriloquist effect (Stekelenburg and Vroomen 2009; Warren et al. 1981). On the other hand, hearing has an advantage in the temporal domain (Burr et al. 2009; Song et al. 2017). One of the essential reasons for the victory of a particular modality is the higher reliability of the information provided by that modality compared to other modalities (Witten and Knudsen 2005).

The visual advantage effect in the training phase seems less stable than that in the testing phase. Specifically, Experiments 2a and 2b showed no modality-based differences in the training phase, but significant modality-based differences in the testing phase. The possible reasons might be that the visual advantage effect occurred in the training phase, but it did not express itself as the occurrence of this effect was a relatively slow process, and the complex visual cues were more challenging to learn. Moreover, one crucial difference between the two phases was that participants in the training phase not only predicted the weather but also learned the association between cues and outcomes based on feedback, while participants in the test phase just needed to predict the weather. It has been demonstrated that the acquired knowledge and the expression of such knowledge can differ across situations (Frensch et al. 1998; Jimenez and Vazquez 2005). Thus, no significant advantage effect in the training phase did not mean that participants had not acquired the category knowledge.

The lack of multisensory facilitation effects in this study may be attributed to several factors. Firstly, attention and working memory resources are assumed to be limited, making it insufficient to complete the classification of all modalities in a short training period (Robinson and Sloutsky 2019). Additionally, most individuals prefer visual information (Pearson 2019), thus prioritizing visual classification. Secondly, when multi-modality facilitation occurs, it is often because the multi-modality provides additionally consistent information compared to a single modality, which means there is information redundancy. In this case, when the single-modality information is already sufficient to complete the task, the cues from other modalities can further enhance the participants’ performance (Girard et al. 2013; Laurienti et al. 2006). However, in the current study, participants must combine information from two dimensions to complete the classification task, and there is no information redundancy. Therefore, the multi-modality information cannot provide additional learning gains.

Moreover, we measured the explicitness of participants’ acquired knowledge using subjective report probabilities and objective betting tasks. The results revealed that participants can acquire both conscious and unconscious category knowledge simultaneously, demonstrating that the WP task involves multiple learning systems. Multiple factors can influence the conscious processing process. First, consciousness is closely related to the stimulus presentation modality. Participants demonstrated explicit or conscious knowledge under the visual modality. However, under the auditory and audiovisual modalities, participants showed varying degrees of unconsciousness. Second, the amount of training influences the acquisition of explicit knowledge. When the training trials extended from 120 trials to 360 trials, the participants primarily acquired conscious knowledge in all three modalities. This finding was consistent with the classical WP task, where the implicit learning system mainly drives early training, while later stages involve a switch to the explicit system (Knowlton et al. 1994, 1996a).

Furthermore, we found that conscious control was not necessary for better performance. Compared to Experiment 1a, auditory feedback (Experiment 2b) and unified auditory stimuli (Experiment 3) both improved conscious knowledge to some extent, but accuracy did not change. However, this improvement was not comprehensive, and awareness control in the auditory and audiovisual modalities was still weaker than in the visual modality. Interestingly, better learning performance was always accompanied by better conscious access. There is evidence of this for both sides. On the one hand, studies focusing on conscious processing on the WP task (Lagnado et al. 2006; Newell et al. 2007; Price 2009; Kemény and Lukács 2013b) revealed that more conscious knowledge is accompanied by better performance. On the other hand, studies in other implicit learning paradigms found a dissociation between performance and conscious access (Miyawaki et al. 2005; Schlaghecken et al. 2000; Zhou et al. 2019, 2020). Thus, this issue could be better explored by controlling for conscious access when comparing across modalities.

The current study also has some limitations. First, we could not completely rule out the possibility that visual stimuli are more accessible to name than auditory stimuli. That is, non-experts can distinguish that a pure tone at 600 Hz is lower than a pure tone at 800 Hz when the two are presented consecutively, but they have a hard time naming sounds like they do colors. When multiple stimuli modalities are mixed, due to the limited capacity of working memory, stimuli that are easier to name are also more likely to emerge in the consciousness. Therefore, future research can use alternative stimuli to investigate this issue. Secondly, this study utilized a modified WP task, in which each cue combination consisted of two cues with a fixed prediction probability. While the experimental paradigm was effective, comparing the results with other studies may be challenging. Consequently, future research could employ a paradigm more closely aligned with the classic WP task. One possibility is maintaining the original probability and combination settings but replacing some cues with audio. Thirdly, due to variations in cue-outcome, correspondences, and cue predictive power, we did not analyze subjects’ strategies in the way used by other studies (e.g., Bochud-Fragnière et al. 2022; Gluck et al. 2002). Future research could analyze categorization strategies to probe further the consciousness of knowledge and subjects’ cognitive processing. Finally, although we have demonstrated that the visual advantage persists in a series of experiments, it is still possible that the visual advantage will disappear when other factors are further manipulated in future works.

In summary, this study utilized the paradigm of probabilistic category learning to investigate the characteristics of unimodality and cross-modality probabilistic category learning. The results reveal that participants can acquire a certain level of category knowledge in visual, auditory, and cross-modality category learning. However, there was a consistent visual advantage effect, where participants had higher optimal response rates and gained more conscious knowledge under visual modalities. Moreover, the knowledge obtained under auditory and cross-modality modalities was primarily unconscious. The results indicate that cue modality could influence the acquisition of category knowledge and the explicitness of the acquired knowledge in probabilistic category learning.

## Figures and Tables

**Table 1:** Cue–outcome correspondence table.

Stimuli No.	Dimension 1	Dimension 2	Optimal Response	Stimuli No.	Dimension 1	Dimension 2	Optimal Response
1	Red	Circle	Rain	7	Wooden fish	Circle	Rain
2	Blue	Rhombus	Rain	8	Guitar	Rhombus	Rain
3	Blue	Circle	Sun	9	Guitar	High tone	Sun
4	Red	Rhombus	Sun	10	Wooden fish	Low tone	Sun
5	Guitar	Circle	Sun	11	Wooden fish	High tone	Rain
6	Wooden fish	Rhombus	Sun	12	Guitar	Low tone	Rain

## Data Availability

Due to participant privacy concerns, the experimental data are currently not publicly available. If there is a legitimate need, please contact the author to obtain the data.
